# Three-Dimensional Choroidal Vessels Assessment in Age-Related Macular Degeneration

**DOI:** 10.1167/iovs.65.13.39

**Published:** 2024-11-19

**Authors:** Elham Sadeghi, Nicola Valsecchi, Mohammed Nasar Ibrahim, Katherine Du, Elli Davis, Sandeep Chandra Bollepalli, Kiran Kumar Vupparaboina, Jose Alain Sahel, Jay Chhablani

**Affiliations:** 1 Department of Ophthalmology, University of Pittsburgh, School of Medicine, Pittsburgh, Pennsylvania, United States; 2 Ophthalmology Unit, Dipartimento di Scienze Mediche e Chirurgiche, Alma Mater Studiorum University of Bologna, Bologna, Italy; 3 IRCCS Azienda Ospedaliero-Universitaria di Bologna, Bologna, Italy; 4 Temple university, School of medicine, Philadelphia, Pennsylvania, United States

**Keywords:** age-related macular degeneration, OCTA, three-dimensional, choroidal vessel, choroidal vascularity index

## Abstract

**Purpose:**

To compare the choroidal vasculature in eyes with early- and intermediate-stage age-related macular degeneration (dAMD) and healthy using a novel three-dimensional algorithm.

**Methods:**

Patients with dAMD and healthy controls underwent clinical examinations and swept-source optical coherence tomography scans (PlexElite-9000 device) centered on the fovea. Scans with quality scores >6 were included. Eyes with any signs of neovascular AMD or geographic atrophy were excluded. The choroidal layer was segmented using ResUNet model and volumetric smoothing. Phansalkar thresholding was used to binarize the choroidal vasculature. The three-dimensional maps were divided into five sectors. The three largest vessels in each sector were measured to determine the mean choroidal vessel diameter (MChVD) and inter-vessel distance (IVD). Volumetric choroidal thickness (ChT) and vascularity index (CVI) were also calculated.

**Results:**

This retrospective cross-sectional study analyzed 60 eyes from 45 dAMD patients (27 early-stage, 33 intermediate-stage) and 26 eyes from 16 healthy controls. The average MChVD was increased in dAMD eyes compared to healthy eyes (239.559 ± 47.058 µm vs. 197.873 ± 49.047 µm, *P* < 0.001). The average MChVD in each sector increased significantly in eyes with dAMD (*P* < 0.05). The average IVD was increased significantly in dAMD eyes compared to healthy eyes (234.128 ± 69.537 µm vs. 179.914 ± 49.995 µm, *P* < 0.001). The average IVD in each sector was significantly increased in eyes with dAMD (*P* < 0.05). Average ChT and CVI in dAMD were reduced compared to healthy eyes (*P* < 0.05).

**Conclusions:**

Eyes with dAMD demonstrated increased MChVD and IVD and decreased ChT and CVI, possibly related to smaller-vessel atrophy and larger-vessel dilation.

Age-related macular degeneration (AMD) is a progressive disease in old age, which starts with drusen and pigmentary changes in the early and intermediate stages and leads to advanced AMD with significant atrophy or new vessel formation affecting central vision.^1^ The underlying causes of the disease remain elusive. Critical neural, structural, and vascular layers are hypothesized to deteriorate, resulting in inflammation, cell autophagy, proteolysis, and lipid accumulation. These changes are thought to stem from abnormal reactions to oxidative stress and inflammation and the triggering of the complement systems.^2^^,^^3^ Retinal pigment epithelium (RPE) and photoreceptors may have a role in AMD pathogenesis.^4^ Additionally, choroidal microvasculature aging significantly contributes to the pathogenesis, resulting in increased vascular resistance and a decrease in choriocapillaris density.^2^^,^^3^

Optical coherence tomography (OCT) is the preferred imaging method for the diagnosis and follow-up of AMD, because it can show the presence of drusens, atrophic changes, and retinal and choroidal thicknesses.^5^ Furthermore, the development of en-face optical coherence tomography (OCT) scans offers rapid spatial mapping of choroidal vessels, enabling a comprehensive analysis of morphological alterations within a single view. Choroidal vascularity index (CVI), which can be generated from volume maps, is a new OCT biomarker used to illustrate choroidal blood vessels in AMD. It is calculated as the ratio of the choroidal vessel volume to the total choroidal volume.^6^^,^^7^ Research suggests that a decreased ratio of the small-vessel layer thickness to the total choroidal thickness, along with an increased thickness of Haller's layer, is associated with a higher incidence of AMD.^8^ Recently it has been shown that choroidal enlarged vessels in the Haller's layer might play a role in the pathogenesis of the disease because they may have a specific distribution pattern of vessel arrangement.^9^ Several studies indicated the Haller layer changes in AMD.^9^^–^^11^ However, choroidal vessel analyses have been done using two-dimensional cross-sectional single or volumetric scans. Choroidal vessels are organized in a more intricate and three-dimensional structure. Thus the three-dimensional (3D) evaluation will provide accurate quantitative analyses.

To address these limitations, our team has formulated a semi-automated algorithm to reconstruct a 3D choroidal vasculature image. Additionally, we have established a method for measuring the diameter of vessels across various segments of the choroid in 3D. Consequently, the primary aim of our current research is to evaluate the largest choroidal vessels that are within the Haller layer in eyes with dry AMD, in comparison to age-matched healthy subjects, by using this innovative 3D approach, and the secondary aim is to compare choroidal vessels between early and intermediate-stage AMD.

## Material and Methods

### Patient Selection

In this retrospective, cross-sectional study, we included the eyes of patients with early and intermediate AMD and healthy age and gender-matched controls. The study was conducted at the Medical Retina and Vitreoretinal Surgery, University of Pittsburgh School of Medicine, from January 2023 to January 2024. The study adhered to the tenets of the Declaration of Helsinki. A “waiver of informed consent” was obtained considering the retrospective nature of the study.

We included patients >55 years old with confirmed diagnosis of early AMD, with small or medium-sized drusen with no pigmentary change, and intermediate-stage AMD, with large drusen with or without pigmentary change, based on the Bechman classification.^12^ Patients with ocular conditions like vitreoretinal diseases, uveitis, glaucoma, retinal vascular diseases, diabetic retinopathy, and high myopia were excluded from the study. Furthermore, individuals who had any ocular surgeries, except for uncomplicated cataract surgeries, were also excluded. Additionally, factors that affected the quality of OCT scans, such as ocular surface disorders, advanced cataracts, or vitreous opacities, were grounds for exclusion. Power analysis was used to determine the minimum sample size required for a study to detect an effect of a given size with a certain level of confidence.

### OCT Imaging Acquisition

Dilated images centered on the fovea were captured using the Plex Elite 9000 device (Carl Zeiss Meditec, Dublin, CA, USA) with an expanded field swept-source (SS-OCT) 12 × 12 mm scan with 100 kHz acquisition. The scan speed of this machine is up to 200,000 A-scans per second, the wavelength is 1060 nm, the scan depth is up to 6 mm in tissue, and the axial resolution is approximately 6.3 µm in tissue. The scan quality was assessed using the SS-OCT software's built-in scoring system, and only scans with scores of 6 or higher (highlighted in green) out of 10 were included in the analysis. SS-OCT scans were exported as eight-bit volumes, each consisting of 1024 B-scans with a resolution of 1024 × 1536. The multimodal imaging acquisitions were subsequently evaluated. An expert retina specialist classified AMD eyes into early and intermediate stages through examination or color fundus photography, following the Beckman classification criteria.^12^

### Automated Choroidal Vessel Segmentation

The outlined procedure involves a combination of automated and manual techniques aimed at measuring the 3D cross-sectional diameter of choroidal vessels. Our technique begins with the application of residual network-based encoder-decoder deep learning architecture (ResUnet) to delineate the choroid's boundaries within all the structural SS-OCT scans.^13^ The process of demarcating the choroid layer is centered on identifying the choroid inner boundary along the junction of the RPE layer and choriocapillaris, and the choroid outer boundary along with the choroidal-scleral junction. A deep learning model followed by a volumetric smoothing process was used for choroidal segmentation, and to avoid potential issues, we used a manual boundary correction. The deep learning model was explained in our previous research studies.^13^^–^^15^ The accuracy of the choroidal boundaries of this algorithm, assessed in unpublished data, was 92.4% in dry AMD eyes, which increased to 100% after manual correction.

The next step in our methodology is the choroidal vasculature segmentation from SS-OCT volumes. According to the challenges with OCT image acquisition, including artifacts such as speckle noise, retinal shadows, contrast fluctuations, volumetric misalignment of B-scans, and complex architecture and intensity characteristics of the choroidal vasculature, we implemented the Phansalkar thresholding method to facilitate the distinction between luminal and stromal areas.^16^^–^^18^

These factors make traditional intensity-based thresholding techniques unsuitable for precise vessel segmentation. To overcome this, we have implemented the Phansalkar thresholding method, a technique recently developed by our group. This method adaptively calculates the local threshold within each 16 × 16 pixel tile across the B-scans, enabling the distinction between luminal and stromal areas.^16^ Subsequently, we apply morphological post-processing to remove extraneous elements, resulting in a seamlessly constructed 3D model of the choroidal vasculature.

**Figure 1.**
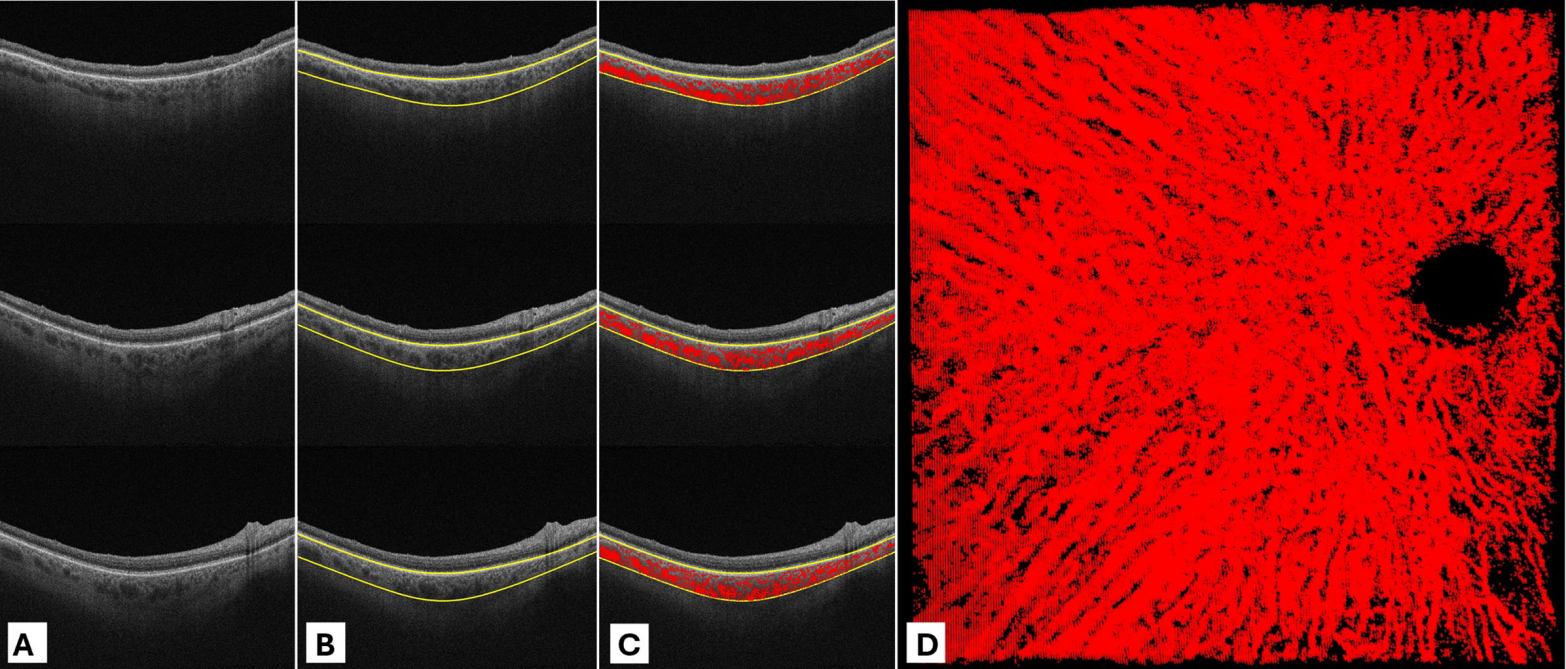
Choroid layer boundary segmentation, automated choroidal vessel segmentation, and three-dimensional choroidal vessels. (**A**) SS-OCT scans from a cube scan of the right eye with an early-stage dry AMD patient. (**B**) Choroidal segmentation in the choroid inner boundary located at the junction of the RPE layer and choriocapillaris, and the choroid outer boundary situated at the choroidal-scleral junction. (**C**) Automated choroidal vessel segmentation. (**D**) The 3D 12 × 12 mm choroidal vessel map with a mask disc. All these steps are done with the first graphical user interface.

We have developed two graphical user interfaces (GUIs) for automated and manual tasks within our proposed method. The first GUI is designed for precise extraction of 3D choroidal vasculature from OCT data, accepting raw SS-OCT volumes in either IMG or JPG formats, segmentation of choroid boundaries, and choroid vessel segmentation. The choroidal segmentation can be corrected manually if needed. As a preparatory step, ImageJ 1.51 s (National Institutes of Health, Bethesda, MD, USA) is used to create an optic disc mask that is used to obscure the choroidal vasculature at optic disc sites. Subsequently, the choroid vessel segmentation is conducted, and the 3D choroidal vasculature models are preserved for manual cross-sectional diameter measurements in the next step (Fig. 1).

**Figure 2.**
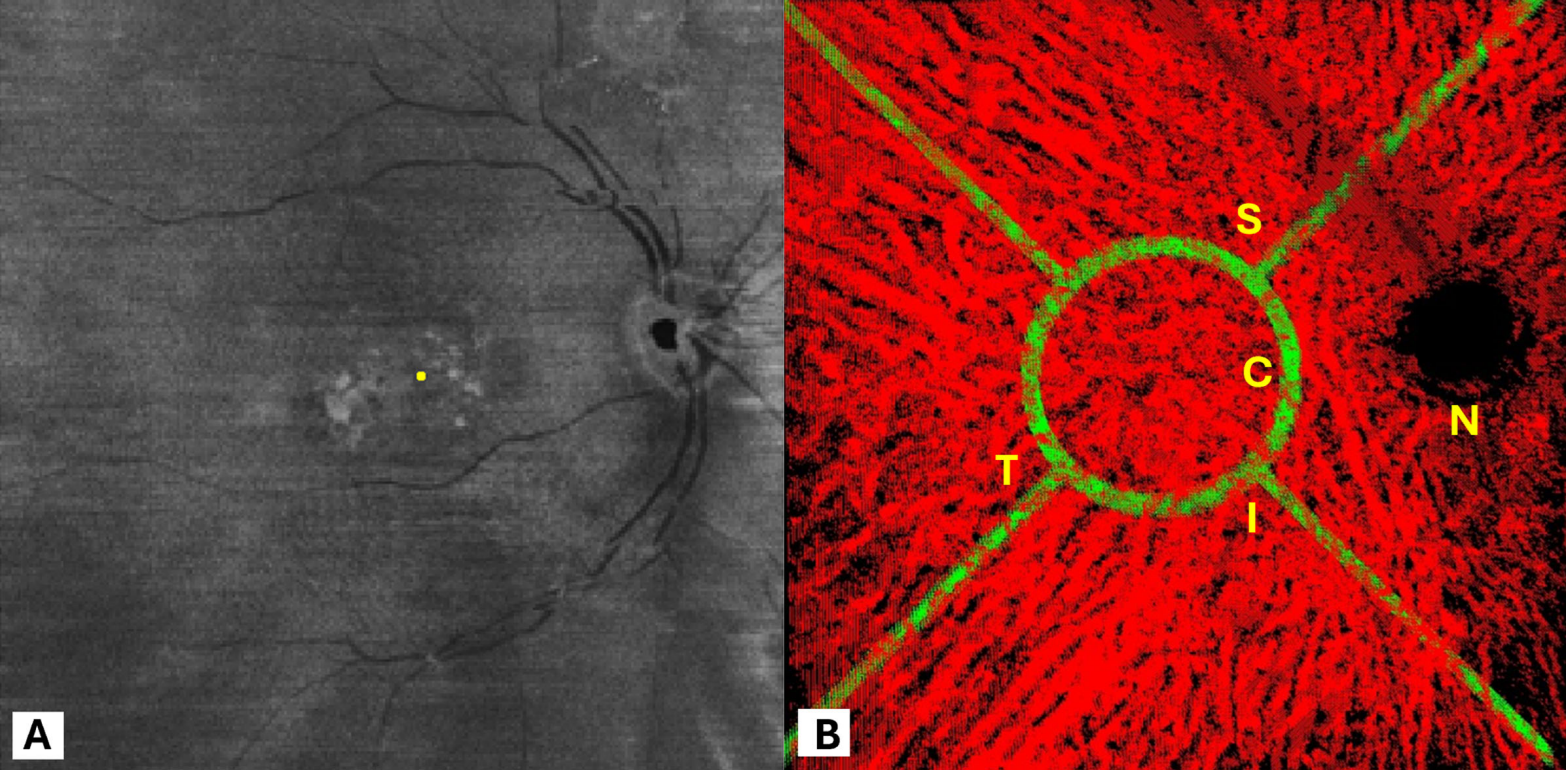
Various sectors including nasal (*N*), temporal (*T*), superior (*S*), inferior (*I*), and central (*C*) in a 3D choroidal vessel map. (**A**) The center of the fovea in the RPE en-face OCT. (**B**) Different sectors based on the center of the fovea. All these steps are done with the second graphical user interface.

To start measuring choroidal thickness, CVI, cross-sectional vessels, and inter-vessel diameters, the datasets are imported into the second GUI, which allows the grader to select any volume. By selecting the center of the fovea in the RPE enface image, which is the center of the foveal avascular zone area in correlation with cross-sectional B scan, a 12 × 12 grid will be applied over the 3D choroidal vasculature, to show different sectors, including central, nasal, temporal, superior, and inferior. The central sector is a circle with a 4 mm diameter. As shown in Figure 2, selecting a point opens a window displaying the vasculature in a small, fixed-size view around that point. The grader can rotate the vasculature to obtain the best view of the vessels and perform measurements more accurately. Simultaneously, the cross-sectional diameter values are recorded in an Excel sheet for subsequent analysis (Fig. 2).

Our algorithm effectively segmented the entire choroid. However, because of the absence of delineation and subsequent reconstruction of the choriocapillaris vessels, the 3D vessel reconstruction is confined to Sattler's and Haller's vessels. This study specifically concentrated on the largest vessels (>100 µm) within each sector, all of which were from the Haller layer.^19^^–^^21^

### Assessment of Choroidal Vessel Diameter and Intervessel Distance

**Figure 3.**
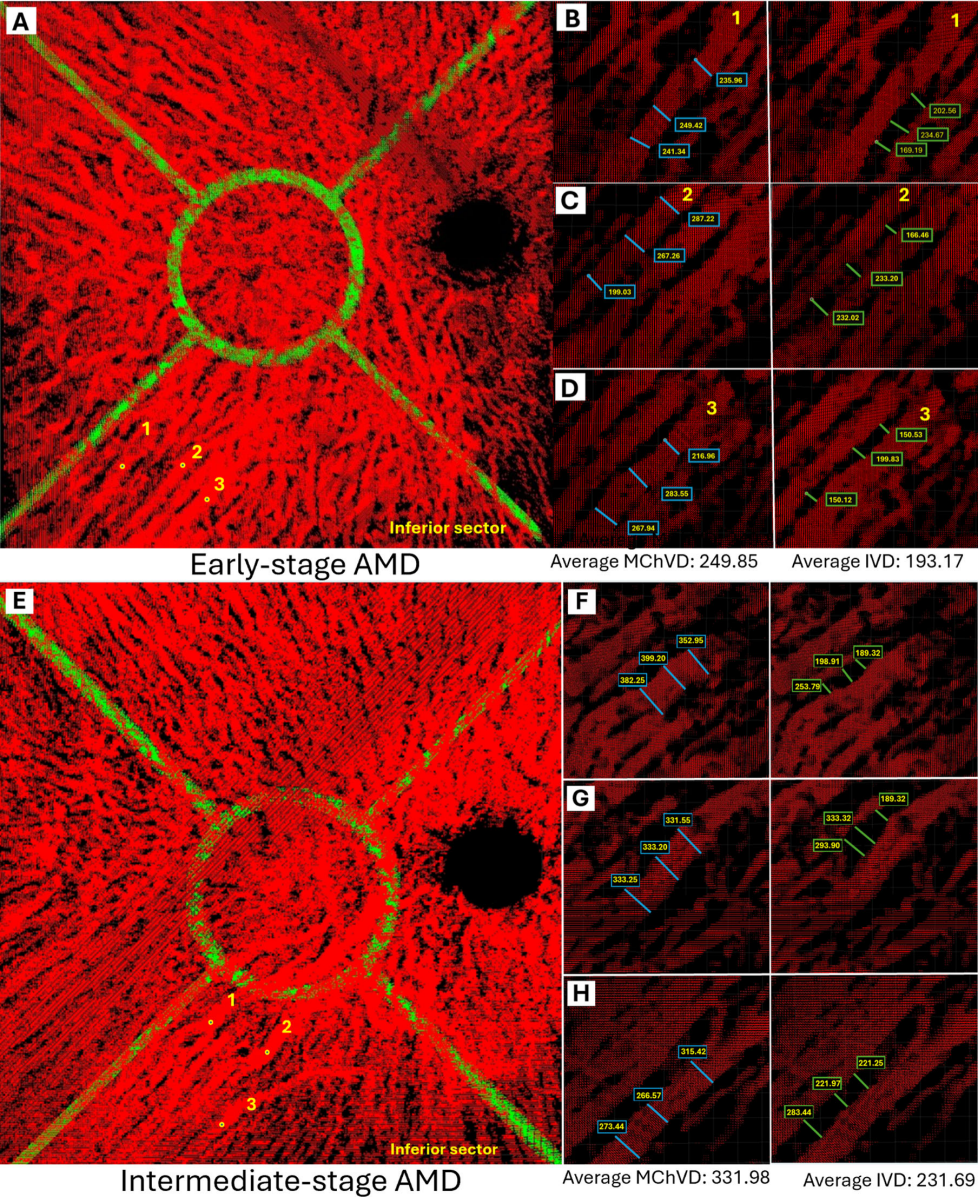
Assessment of MChVD and IVD in the inferior sector of the right eye in early- and intermediate-stage AMD. The three largest vessels from the inferior sector are labeled as *1*, *2*, and *3*. (**A**) These vessels were then oriented for a detailed diameter assessment. The cross-sectional diameter was measured from the outermost visible portions of each vessel, with three measurements taken per vessel (*blue border*). The IVD was determined by measuring the distance between the largest selected vessel and the nearest independent vessel (*green border*). Vessel 1 is shown in **B**, vessel 2 in **C**, and vessel 3 in **D**. The mean of the nine diameters was calculated to represent the MChVD of the inferior sector, and the mean of the nine IVD measurements was calculated to represent the mean IVD in the inferior sector. Values are reported in micrometers. The same procedure is applied to **E–H** as an intermediate-stage AMD. Vessel 1 is shown in **F**, vessel 2 in **G**, and vessel 3 in **H**, and MChVD (*blue border*) and IVD (*green border*) are measured. All these steps are done with the second graphical user interface.

The analysis entailed the selection of the three largest vessels within each sector, with three measurements recorded for each vessel. Before the selection process, the images were rotated to obtain a comprehensive 3D perspective, facilitating the identification of the three largest vessels per sector. Subsequently, the vessels were oriented to allow for a meticulous assessment of their diameters. The cross-sectional diameter was measured from the outermost visible portions of each vessel, with three measurements taken for each vessel. The average of these nine measurements (three per vessel) was then analyzed to determine the average choroidal vessel diameter (MChVD) for each sector. We defined the IVD as the space separating the largest vessel (which we evaluated for the MChVD) from the closest non-collateral vessel within the same sector. The average of these nine measurements (three per vessel) was then analyzed to determine the average IVD for each sector (Fig. 3).

To evaluate the intraclass correlation coefficient (ICC) test, two masked readers, blinded to the patient's details, performed measurements across all sectors for ten eyes (E.S., N.V.). The measurements taken by the first grader (E.S.) were used for the study analysis, whereas the second grader (N.V.) measurements were used to evaluate intergrader reliability. The choroidal thickness (ChT) and the CVI for the entire volume were determined using specialized automated software.

### Statistical Analysis

The assessment of data normality was conducted using the Shapiro–Wilk test, followed by the application of parametric tests. To evaluate the consistency between raters for image binarization, we used the absolute agreement model of the ICC. The ICC values were interpreted as follows: less than 0.5 indicated poor reliability, between 0.5 and 0.75 suggested moderate reliability, between 0.75 and 0.9 denoted good reliability, and values greater than 0.90 signified excellent reliability. For categorical data analysis, the χ^2^ test was used. Demographic data, ChT, CVI, mean MChVD, and IVD were compared across different groups using linear mixed models. These groups included early and intermediate stages of dry AMD versus age- and gender-matched healthy patients. We also compare these parameters between early AMD and intermediate AMD eyes and between healthy and early-stage AMD. A *P* value <0.05 was used to indicate statistical significance. All statistical analyses were conducted using IBM's Statistical Package for Social Sciences (SPSS) version 26.

## Results

### Demographic Data

A total of 86 eyes of 61 individuals were included in our analysis (60 eyes of 45 AMD patients, and 26 eyes of 16 healthy age-gender-matched controls). Among the 45 patients in the AMD group, 15 exhibited bilateral dAMD, with both eyes included in the study. Of these, eight had bilateral early-stage AMD, and six had bilateral intermediate-stage AMD. One patient had early AMD in the right eye and intermediate-stage AMD in the left eye. In 11 patients with dAMD, only one eye was included because of low-quality OCT angiography (OCTA) in the other eye. In 19 patients, only one eye was included because of neovascular AMD in the other eye. Among these, four patients had early-stage AMD in the included eye, whereas 14 had intermediate-stage AMD. Among the 16 subjects in the healthy group, both eyes were included in 10 cases. However, for six subjects, only one eye was included because of poor-quality OCTA in the excluded eye.

Overall, the mean age of patients was 75.40 ± 9.55 years, and 39 (63.90%) were females. No differences in age were observed between the two groups (76.48 ± 9.07 years in AMD vs. 72.37 ± 8.42 years in healthy patients, *P* = 0.140). Best-corrected visual acuity (BCVA) was diminished in AMD eyes compared to controls (0.150 ± 0.160 vs 0.016 ± 0.038 LogMAR, *P* < 0.001). Of all the dry AMD eyes, 27 (45.00%) were in the early stage, and 33 (55.00%) were in the intermediate stage. The mean BCVA was lower in intermediate AMD compared to early AMD (0.163 ± 0.169 vs. 0.096 ± 0.133 LogMAR, *P* = 0.022; Table 1). Assessment of ten eyes for ICC between two masked readers for measurements of MChVD and IVD showed a high degree of agreement across all sectors, with an ICC value of 0.887 and a confidence interval of 0.806 to 0.928.

**Table 1.** Demographic Data

	Total 86 Eyes, 61 Subjects	AMD 60 Eyes, 45 Patients	Healthy 26 Eyes, 16 Subjects	*P* Value
Age (year), Mean ± SD	75.40 ± 9.55	76.48 ± 9.07 (range 55-95)	72.37 ± 8.42 (range 60-84)	0.140
Female	39 (63.90%)	28 (62.20%)	10 (62.50%)	0.647
Right eye	35 (50.00%)	27 (45.00%)	14 (53.84%)	0.734
BCVA (LogMAR), Mean ± SD	0.07 ± 0.128	0.150 ± 0.160	0.016 ± 0.038	**<0.001**

Bold face entries indicate *P* values < 0.05.

### ICC in 3D Assessment

#### 3D Assessment in AMD and Healthy Eyes

The average ChT was found to be significantly reduced in eyes with dry AMD when compared to the healthy group (210.809 ± 68.446 µm vs. 236.337 ± 62.734 µm, *P* < 0.001). The ChT in all sectors was lower in AMD eyes compared to the healthy group, as shown in Table 2.

**Table 2.** A Comparison of Choroidal Parameters Such as MChVD, IVD, ChT, and CVI

	Dry AMD 60 Eyes of 45 Patients	Healthy 26 Eyes of 16 Patients	*P* Value	Confidence Interval	Beta Coefficient
MChVD (µm)					
Average	239.559 ± 47.058	197.873 ± 49.047	**<0.001**	186.019, 213.180	199.599
Nasal	230.593 ± 50.797	198.748 ± 62.248	**0.015**	176.651, 222.662	199.657
Temporal	228.015 ± 47.491	183.552 ± 42.552	**<0.001**	164.026, 204.741	184.383
Inferior	241.926 ± 53.579	210.500 ± 49.697	**0.014**	189.153, 231.793	210.473
Superior	246.408 ± 43.087	202.405 ± 39.735	**<0.001**	185.013, 222.270	203.641
Central	227.516 ± 42.386	194.643 ± 47.705	**0.002**	176.065, 215.537	195.801
IVD (µm)					
Average	234.128 ± 69.537	179.914 ± 49.995	**<0.001**	163.213, 198.511	180.862
Nasal	237.162 ± 67.093	176.223 ± 57.553	**<0.001**	151.057, 201.390	176.224
Temporal	229.208 ± 63.023	180.136 ± 49.763	**<0.001**	156.975, 203.297	180.136
Inferior	230.958 ± 66.955	178.668 ± 45.324	**<0.001**	152.652, 205.273	178.962
Superior	239.215 ± 64.991	195.633 ± 52.444	**0.003**	169.050, 221.514	195.282
Central	231.991 ± 76.535	168.861 ± 43.432	**<0.001**	140.526, 199.527	170.027
ChT (µm)					
Average	210.809 ± 68.446	236.337 ± 62.734	**<0.001**	206.343, 263.743	235.043
Nasal	176.204 ± 55.800	214.431 ± 60.283	**0.006**	183.627, 240.425	212.026
Temporal	205.357 ± 55.464	228.263 ± 49.575	0.071	200.740, 255.673	228.207
Inferior	218.179 ± 67.585	259.703 ± 68.329	**0.012**	222.190, 290.766	256.478
Superior	182.363 ± 59.744	221.973 ± 60.284	**0.006**	190.481, 250.622	220.551
Central	232.251 ± 83.624	258.214 ± 64.162	0.162	217.343, 297.407	257.375
CVI (%)					
Average	0.368 ± 0.045	0.368 ± 0.042	**0.002**	0.367, 0.403	0.385
Nasal	0.329 ± 0.054	0.368 ± 0.041	**0.002**	0.344, 0.394	0.369
Temporal	0.348 ± 0.044	0.366 ± 0.034	0.073	0.347, 0.388	0.368
Inferior	0.380 ± 0.034	0.395 ± 0.020	**0.048**	0.381, 0.414	0.398
Superior	0.366 ± 0.048	0.398 ± 0.035	**0.004**	0.379, 0.422	0.400
Central	0.367 ± 0.051	0.387 ± 0.031	0.066	0.367, 0.414	0.391

Bold face entries indicate *P* values < 0.05.

These measurements are detailed for the average area, as well as for all the sectors separately. The table contrasts these parameters between eyes affected by dry AMD and those that are healthy.

The average CVI showed a reduction in eyes with dry AMD relative to healthy eyes (0.368 ± 0.045 vs. 0.368 ± 0.042, *P* = 0.002). The CVI values were consistently lower in the AMD group in all the sectors, and the values are shown in Table 2.

The average MChVD in all the sectors (three vessels per sector) was increased in AMD eyes compared to healthy eyes (239.559 ± 47.058 vs. 197.873 ± 49.047 µm, *P* < 0.001). By evaluating the MChVD in each sector, we found that eyes with dry AMD exhibited an increase in MChVD in each sector, with a statistically significant difference compared to healthy eyes.

The average IVD was significantly increased in AMD eyes compared to healthy eyes (234.128 ± 69.537 µm vs. 179.914 ± 49.995 µm, *P* < 0.001). By analyzing the mean IVD in each sector, we found a significant increase in eyes with dry AMD in comparison to healthy eyes in each sector (Table 2). A representative example is shown in Figure 3.

#### 3D Assessment in Early Versus Intermediate AMD

Of all the dry AMD eyes, 27 (45.00%) were classified as early-stage, whereas 33 (55.00%) were defined as intermediate-stage. The average ChT across all sectors was lower in the intermediate-stage AMD group compared to the early-stage group (215.606 ± 83.592 µm vs. 192.738 ± 51.142 µm, *P* = 0.411). On sector-by-sector analysis, the ChT was consistently lower in the intermediate-stage group, but the results were not statistically significant (*P* > 0.05).

The average CVI across all sectors was found to be lower in eyes with intermediate-stage dry AMD compared to those in the early stage (0.367 ± 0.056 vs. 0.351 ± 0.044, *P* = 0.703). On sector-by-sector analysis, the CVI was consistently lower in the intermediate-stage group; however, these variations were not statistically significant (*P* > 0.05).

The average MChVD for all sectors, considering three vessels per sector, revealed no significant distinction between the early and intermediate stages of dry AMD (early-stage: 232.874 ± 45.487 µm vs. intermediate-stage: 236.542 ± 50.006 µm, *P* = 0.255). Additionally, when examining each sector individually, the differences in MChVD remained statistically nonsignificant (*P* > 0.05). The average IVD of all the sectors did not show a significant difference between early and intermediate-stage AMD eyes (early stage: 240.870 ± 71.638 µm vs intermediate stage: 227.847 ± 63.664 µm, *P* = 0.169). Furthermore, when assessing the IVD within each sector, no significant differences were observed (*P* > 0.05) (Table 3). The comparison between healthy eyes and those with early AMD revealed a statistically significant increase in average MChVD (*P* = 0.003), particularly in the temporal (*P* = 0.002) and superior sectors (*P* < 0.001). Although the nasal, temporal, and central sectors exhibited a trend toward higher MChVD (*P* > 0.05), this was not statistically significant. Eyes with early-stage AMD demonstrated significantly higher IVD across all sectors (*P* < 0.05) and a trend toward lower ChT in all sectors (*P* > 0.05). Additionally, CVI showed a decreasing trend in eyes with early-stage AMD, with a statistically significant reduction in the nasal sector (*P* = 0.003) (Table 4).

**Table 3.** A Comparison of Choroidal Parameters Such as MChVD

	Early-Stage AMD 27 Eyes (45.00%)	Intermediate-Stage AMD 33 Eyes (55.00%)	*P* Value	Confidence Interval	Beta Coefficient
MChVD (µm)					
Average	232.874 ± 45.487	236.542 ± 50.006	0.255	227.942, 248.749	238.345
Nasal	226.083 ± 41.834	234.282 ± 56.472	0.538	216.489, 252.077	234.283
Temporal	226.541 ± 45.888	229.221 ± 48.438	0.359	208.719,244.089	226.404
Inferior	231.523 ± 48.299	250.438 ± 56.846	0.157	231.587, 269.573	250.580
Superior	252.178 ± 43.533	241.686 ± 42.802	0.294	225.511, 257.187	241.349
Central	228.046 ± 43.994	227.083 ± 41.706	0.344	210.206, 241.715	225.961
IVD (µm)					
Average	240.870 ± 71.638	227.847 ± 63.664	0.169	214.270, 244.244	229.257
Nasal	238.781 ± 69.829	235.838 ± 66.160	0.868	212.203, 259.474	235.839
Temporal	226.803 ± 46.683	231.176 ± 74.456	0.792	209.040, 253.312	231.176
Inferior	243.494 ± 73.710	220.701 ± 60.083	0.072	197.516, 243.887	220.701
Superior	253.014 ± 66.381	227.926 ± 62.572	0.134	205.086, 251.031	228.059
Central	242.258 ± 95.824	223.592 ± 56.371	0.456	196.159, 251.300	223.729
ChT (µm)					
Average	215.606 ± 83.592	192.738 ± 51.142	0.411	168.737, 206.335	187.536
Nasal	188.980 ± 67.991	166.138 ± 42.366	0.313	144.446, 187.056	165.751
Temporal	215.383 ± 75.473	196.965 ± 31.168	0.274	174.182, 217.790	195.986
Inferior	237.048 ± 85.434	203.312 ± 45.382	0.103	175.946, 226.199	201.073
Superior	187.081 ± 74.614	178.646 ± 45.690	0.732	154.319, 201.061	177.690
Central	249.540 ± 98.039	218.630 ± 68.785	0.292	183.981, 248.037	216.009
CVI (%)					
Average	0.367 ± 0.056	0.351 ± 0.044	0.703	0.340, 0.367	0.354
Nasal	0.336 ± 0.063	0.323 ± 0.047	0.756	0.301, 0.344	0.323
Temporal	0.361 ± 0.053	0.339 ± 0.034	0.139	0.320, 0.354	0.337
Inferior	0.392 ± 0.033	0.371 ± 0.033	0.138	0.358, 0.385	0.372
Superior	0.377 ± 0.062	0.359 ± 0.034	0.556	0.339, 0.376	0.357
Central	0.368 ± 0.054	0.366 ± 0.050	0.733	0.343, 0.384	0.364

IVD, ChT, and CVI are detailed for the area, as well as for all the sectors separately. The table contrasts these parameters between eyes affected by early-stage AMD and intermediate-stage AMD eyes.

**Table 4.** Comparison of Choroidal Parameters

	Early-Stage AMD, 27 Eyes	Healthy, 26 Eyes	*P* Value	Confidence Interval	Beta Coefficient
MChVD (µm)					
Average	232.874 ± 45.487	197.873 ± 49.047	**0.003**	185.169, 214.544	199.857
Nasal	226.083 ± 41.834	198.748 ± 62.248	0.099	176.584, 223,601	200.092
Temporal	226.541 ± 45.888	183.552 ± 42.552	**0.002**	165.330, 202.234	183.782
Inferior	231.523 ± 48.299	210.500 ± 49.697	0.128	190.827, 230.175	210.501
Superior	252.178 ± 43.533	202.405 ± 39.735	**<0.001**	184.493, 223.790	204.141
Central	228.046 ± 43.994	194.643 ± 47.705	0.070	174.817, 217.381	196.099
IVD (µm)					
Average	240.870 ± 71.638	179.914 ± 49.995	**<0.001**	161.200, 200.894	181.047
Nasal	238.781 ± 69.829	176.223 ± 57.553	**0.003**	149.002, 203.845	176.423
Temporal	226.803 ± 46.683	180.136 ± 49.763	**<0.001**	161.152, 199.120	180.136
Inferior	243.494 ± 73.710	178.668 ± 45.324	**0.002**	151.685, 206.441	179.063
Superior	253.014 ± 66.381	195.633 ± 52.444	**0.005**	168.097, 222.044	195.071
Central	242.258 ± 95.824	168.861 ± 43.432	**0.004**	137.109, 203.030	170.070
ChT (µm)					
Average	215.606 ± 83.592	236.337 ± 62.734	0.418	199.160, 270.857	235.008
Nasal	188.980 ± 67.991	214.431 ± 60.283	0.275	179.157 ± 244.818	211.988
Temporal	215.383 ± 75.473	228.263 ± 49.575	0.662	193.812, 262.595	228.203
Inferior	237.048 ± 85.434	259.703 ± 68.329	0.428	216.007, 296.926	256.466
Superior	187.081 ± 74.614	221.973 ± 60.284	0.168	185.696, 255.421	220.558
Central	249.540 ± 98.039	258.214 ± 82.151	0.773	213.324, 301.427	257.376
CVI (%)					
Average	0.367 ± 0.056	0383 ± 0.035	0.087	0.365, 0.406	0.385
Nasal	0.336 ± 0.063	0.368 ± 0.041	**0.003**	0.341, 0.396	0.369
Temporal	0.361 ± 0.053	0.366 ± 0.034	0.618	0.344, 0.391	0.368
Inferior	0.392 ± 0.033	0.395 ± 0.033	0.492	0.383, 0.412	0.398
Superior	0.377 ± 0.062	0.398 ± 0.035	0.104	0.375, 0.425	0.400
Central	0.368 ± 0.054	0.387 ± 0.031	0.069	0.367, 0.415	0.391

Bold face entries indicate *P* values < 0.05.

IVD, ChT, CVI are detailed for the area and for all the sectors separately. The table contrasts these parameters between eyes affected by early-stage AMD and healthy eyes.

## Discussion

By using an innovative 3D algorithm to evaluate choroidal vessels, our study found that the average MChVD was increased in dry AMD eyes compared to age-matched healthy eyes (*P* < 0.001). We showed that the average MChVD in each sector increased significantly in eyes with dry AMD (*P* < 0.05). Also, the results revealed that the mean IVD was significantly increased in dry AMD eyes compared to healthy eyes (*P* < 0.001). The average IVD in each sector was significantly increased in eyes with dry AMD (*P* < 0.05). Additionally, this study exhibited a reduction in average ChT in dry AMD compared to healthy eyes (*P* < 0.001) and a reduction in average ChT in eyes with dry AMD per each sector (*P* > 0.05). In comparing healthy and early-stage AMD, the eyes with early-stage AMD showed significantly higher MChVD and IVD (*P* > 0.05). Furthermore, we observed that the eyes at the intermediate stage of AMD showed reduced ChT and CVI in all sectors compared to those at the early stage, although statistical significance was not reached (*P* > 0.05).

In our research, we implemented an advanced semi-automated algorithm designed to generate 3D visualizations of choroidal vessels. This innovative tool enabled us to accurately measure the diameters of these vessels and the distances between larger ones within a 3D framework. Consequently, we were able to conduct a comprehensive analysis of the choroidal vasculature, choroidal stroma, and their spatial dynamics in patients with early and intermediate stages of dry AMD. Prior research predominantly concentrated on analyzing choroidal biomarkers through OCT B-scan or volumetric reconstructions of the choroidal vasculature.^22^ Nevertheless, such methodologies fall short of delivering an exhaustive evaluation of the choroidal vasculature, including assessments of vessel diameters and IVD, which are uniquely ascertainable through 3D imaging techniques. Considering the variable depths, measurements obtained on two-dimensional imaging or en-face imaging are unreliable. Thus 3D analysis is essential for choroidal vessel analysis.

The deterioration of choriocapillaris endothelial cells, RPE, and photoreceptors significantly contribute to AMD onset.^4^^,^^23^ Several studies indicated that a reduced ratio of the small-vessel layer's thickness to the choroid's average thickness, coupled with an increased thickness in Haller's layer, correlates with a greater incidence of AMD. ^8^ An en-face SS-OCT study by Baek et al.^24^ has found large choroidal vessels in 46% of cases with neovascular AMD, highlighting that morphological alterations extend beyond the choriocapillaris to the deeper choroidal vessels. These findings include a general thinning of the choroid with localized dilations in the deeper vessels. Furthermore, Sacconi et al.^25^ have identified potential circulatory pattern anomalies within Haller's layer in patients with dry AMD including laterally diagonal and reticular patterns, suggesting that abnormalities in the choroidal enlarged vessels may contribute to the disease's development. Consistent with prior research, our findings indicate an enlargement of the MChVD in eyes affected by dry AMD. These findings suggest that the pathogenesis of AMD from RPE, photoreceptor, and choriocapillaris extends its impact to the larger vessels of the choroid as well. Besides vessel diameter changes, changes in the choroidal interstitial stroma were histologically reported in eyes affected by AMD; In the disease progression, there is a sequential degeneration of the choroidal vasculature with subsequent stromal and fibrous tissue replacement.^26^^,^^27^ Our study observed an increase in the IVD in a 3D perspective, potentially suggesting that this biomarker might serve as a measure of the choroidal stroma situated among the large Haller's vessels, which may be attributed to smaller vessel degeneration and replacement with fibrosis and stroma. The observed reduction in ChT, coupled with an increase in MChVD and IVD, substantiate this discussion.

CVI, as a novel parameter derived from OCT imaging showing a ratio of choroidal vessel volume to choroidal stroma, has been suggested to effectively measure choroidal structural alterations in various disorders, including AMD.^28^ A decline in CVI may indicate a secondary consequence of AMD or implicate choroidal ischemia in its pathogenesis.^28^ It is associated with different degrees of AMD progression risk, and it could be used as a biomarker of AMD progression.^27^ It is typically measured using a single two-dimensional OCT B-scan with binarization.^6^ The assessment of CVI in macular diseases has enriched our comprehension of potential structural alterations. In our analysis, we utilized three-dimensional imaging to evaluate CVI, providing a more detailed assessment. Our study demonstrated a trend of decreasing CVI among AMD patients compared to healthy controls and also when comparing intermediate-stage AMD to early-stage AMD across all evaluated sectors. Although the CVI changes from the early to intermediate stage were not significant, the trend of regression shows slight changes during the disease progression.

During the disease progression, there is a notable choroidal thinning and CVI reduction. These biomarkers are valuable for assessing the risk of disease progression and predicting visual acuity outcomes. Additionally, the possibility of medium-sized vessels being replaced with fibrotic tissue may lead to compensatory dilation of the remaining vessels, evidenced by the increase in the diameter of large vessels. Alterations in choroidal vessel diameter and intervessel distance could thus serve as novel biomarkers for AMD progression.

The limitations of our study are its retrospective nature and small sample size. Although we considered power analysis, more sample size may help to explore more details. Because of the retrospective cross-sectional design of our study, tracking the progressive changes in AMD patients over time, which is vital for understanding the evolution of choroidal changes, was not feasible. Another limitation of our study is the absence of axial length correction for OCTA scales. Although the use of angular units, such as arcminutes or degrees, would have been preferable to linear units, we were unable to implement this adjustment.^29^^,^^30^ Our algorithm successfully segmented the whole choroid; however, because of the lack of delineation and subsequent reconstruction of the choriocapillaris vessels, the 3D vessel reconstruction is limited to Sattler's and Haller's vessels. This study specifically focused on the largest vessel (>100 µm) within each sector, all of which were from the Haller layer.^19^^–^^21^ To gain deeper insights into choroidal changes in advanced AMD cases, including those with geographic atrophy and neovascular AMD, additional research examining 3D images is essential, which is our team's future goal.

## Conclusions

In the eyes affected by dry AMD, there was a notable increase in average MChVD and IVD in all the sectors. Concurrently, there was a significant reduction in the average CVI and ChT when compared to healthy eyes. Additionally, eyes in the intermediate-stages AMD showed a trend of lower ChT and CVI values than those in the early stage. Overall, the application of three-dimensional choroidal imaging presents a unique noninvasive approach for investigating choroidal changes in dry AMD and other eye diseases and may potentially enhance our grasp of the underlying mechanisms driving their pathogenesis. Future research could enable automated measurements of vessel diameters and IVD, as well as assessments of the positioning and orientation of choroidal vessels in 3D images in all stages of AMD. These measurements could serve as imaging markers of disease progression, helping to identify patients at risk.

## References

[1] Shome I, Thathapudi NC, Aramati BM, Kowtharapu BS, Jangamreddy JR. Stages, pathogenesis, clinical management and advancements in therapies of age-related macular degeneration. *Int Ophthalmol*. 2023; 43: 3891–3909. 37347455 10.1007/s10792-023-02767-2

[2] Fleckenstein M, Schmitz-Valckenberg S, Chakravarthy U. Age-related macular degeneration: a review. *JAMA*. 2024; 331: 147–157. 38193957 10.1001/jama.2023.26074 PMC12935482

[3] Garcia-Garcia J, Usategui-Martin R, Sanabria MR, Fernandez-Perez E, Telleria JJ, Coco-Martin RM. Pathophysiology of age-related macular degeneration: implications for treatment. *Ophthalmic Res*. 2022; 65: 615–636. 35613547 10.1159/000524942

[4] Nowak JZ . Age-related macular degeneration (AMD): pathogenesis and therapy. *Pharmacol Rep*. 2006; 58: 353. 16845209

[5] Vallino V, Berni A, Coletto A, et al. Structural OCT and OCT angiography biomarkers associated with the development and progression of geographic atrophy in AMD. *Graefes Arch Clin Exp Ophthalmol*. 2024: 1–6. 38689123 10.1007/s00417-024-06497-8 PMC11584504

[6] Agrawal R, Ding J, Sen P, et al. Exploring choroidal angioarchitecture in health and disease using choroidal vascularity index. *Prog Retinal Eye Res*. 2020; 77: 100829. 10.1016/j.preteyeres.2020.100829 31927136

[7] Corbelli E, Sacconi R, Battista M, et al. Choroidal vascularity index in eyes with central macular atrophy secondary to age-related macular degeneration and Stargardt disease. *Graefes Arch Clin Exp Ophthalmol*. 2022; 260: 1525–1534. 35048199 10.1007/s00417-021-05337-3

[8] Zhao J, Wang YX, Zhang Q, Wei WB, Xu L, Jonas JB. Macular choroidal small-vessel layer, Sattler's layer and Haller's layer thicknesses: the Beijing Eye Study. *Sci Rep*. 2018; 8(1): 4411. 29535365 10.1038/s41598-018-22745-4 PMC5849687

[9] Sacconi R, Cicinelli MV, Borrelli E, et al. Haller's vessels patterns in non-neovascular age-related macular degeneration. *Graefes Arch Clin Exp Ophthalmol*. 2020; 258: 2163–2171. 32535671 10.1007/s00417-020-04769-7

[10] Kim S, Lee H, Chung H, Kim HC. Choroidal neovascularization and Haller vessel morphology associated with vision and treatment number after 1 year in age-related macular degeneration. *Korean J Ophthalmol*. 2021; 35: 397–409. 34344131 10.3341/kjo.2021.0085 PMC8521327

[11] Hacker V, Reiter GS, Schranz M, et al. Impact of large choroidal vessels on choriocapillaris flow deficit analyses in optical coherence tomography angiography. *PloS One*. 2021; 16(8): e0254955. 34343177 10.1371/journal.pone.0254955 PMC8330935

[12] Ferris FL III, Wilkinson CP, Bird A, et al. Clinical classification of age-related macular degeneration. *Ophthalmology*. 2013; 120: 844–851. 23332590 10.1016/j.ophtha.2012.10.036 PMC11551519

[13] Arora S, Singh SR, Rosario B, et al. Three-dimensional choroidal contour mapping in healthy population. *Sci Rep*. 2024; 14(1): 6210. 38485744 10.1038/s41598-024-56376-9 PMC10940280

[14] Vupparaboina KK, Selvam A, Suthaharan S, et al. Automated choroid layer segmentation based on wide-field SS-OCT images using deep residual encoder-decoder architecture. *Invest Ophthalmol Vis Sci*. 2021; 62: 2162–2162.

[15] Ibrahim MN, Bashar SB, Rasheed MA, et al. Volumetric quantification of choroid and Haller's sublayer using OCT scans: an accurate and unified approach based on stratified smoothing. *Comput Med Imaging Graph*. 2022; 99: 102086. 35717830 10.1016/j.compmedimag.2022.102086 PMC13464413

[16] Ibrahim M, Bollepalli SC, Selvam A, et al. Accurate detection of 3D choroidal vasculature using swept-source OCT volumetric scans based on Phansalkar thresholding. In: 2023 IEEE EMBS International Conference on Biomedical and Health Informatics (BHI). IEEE. 2023.

[17] Agrawal R, Seen S, Vaishnavi S, et al. Choroidal vascularity index using swept-source and spectral-domain optical coherence tomography: a comparative study. *Ophthalmic Surg Lasers Imaging Retina*. 2019; 50(2): e26–e32. 30768226 10.3928/23258160-20190129-15

[18] Vupparaboina KK, Richhariya A, Chhablani JK, Jana S. Optical coherence tomography imaging: automated binarization of choroid for stromal-luminal analysis. 2016 International Conference on Signal and Information Processing (IConSIP), 2016;1–5.

[19] Shiihara H, Seen S, Vaishnavi S, et al. Quantification of vessels of Haller's layer based on en-face optical coherence tomography images. *Retina*. 2021; 41: 2148–2156. 33734190 10.1097/IAE.0000000000003166

[20] Esmaeelpour M, Kajic V, Zabihian B, et al. Choroidal Haller's and Sattler's layer thickness measurement using 3-dimensional 1060-nm optical coherence tomography. *PloS One*. 2014; 9(6): e99690. 24911446 10.1371/journal.pone.0099690 PMC4050051

[21] Singh SR, Vupparaboina KK, Goud A, Dansingani KK, Chhablani J. Choroidal imaging biomarkers. *Surv Ophthalmol*. 2019; 64: 312–333. 30496750 10.1016/j.survophthal.2018.11.002

[22] Breher K, Terry L, Bower T, Wahl S. Choroidal biomarkers: a repeatability and topographical comparison of choroidal thickness and choroidal vascularity index in healthy eyes. *Transl Vis Sci Technol*. 2020; 9(11): 8–8. 10.1167/tvst.9.11.8 PMC7552934 33133771

[23] Scholfield C, McGeown J, Curtis T. Cellular physiology of retinal and choroidal arteriolar smooth muscle cells. *Microcirculation*. 2007; 14: 11–24. 17365658 10.1080/10739680601072115

[24] Baek J, Lee JH, Jung BJ, Kook L, Lee WK. Morphologic features of large choroidal vessel layer: age-related macular degeneration, polypoidal choroidal vasculopathy, and central serous chorioretinopathy. *Graefes Arch Clin Exp Ophthalmol*. 2018; 256: 2309–2317. 30259090 10.1007/s00417-018-4143-1

[25] Sacconi R, Cicinelli MV, Borrelli E, et al. Haller's vessels patterns in non-neovascular age-related macular degeneration. *Graefes Arch Clin Exp Ophthalmol*. 2020; 258: 2163–2171. 32535671 10.1007/s00417-020-04769-7

[26] McLeod DS, Grebe R, Bhutto I, Merges C, Baba T, Lutty GA. Relationship between RPE and choriocapillaris in age-related macular degeneration. *Invest Ophthalmol Vis Sci*. 2009; 50: 4982–4991. 19357355 10.1167/iovs.09-3639 PMC4829357

[27] Sacconi R, Vella G, Battista M, et al. Choroidal vascularity index in different cohorts of dry age-related macular degeneration. *Transl Vis Sci Technol*. 2021; 10(12): 26–26. 10.1167/tvst.10.12.26 PMC8543393 34665234

[28] Wei X, Ting DS, Ng WY, et al. Choroidal vascularity index: a novel optical coherence tomography based parameter in patients with exudative age-related macular degeneration. *Retina*. 2017; 37: 1120–1125. 27632714 10.1097/IAE.0000000000001312

[29] Linderman R, Salmon AE, Strampe M, Russillo M, Khan J, Carroll J. Assessing the accuracy of foveal avascular zone measurements using optical coherence tomography angiography: segmentation and scaling. *Transl Vis Sci Technol*. 2017; 6(3): 16. 10.1167/tvst.6.3.16 PMC5469394 28616362

[30] Llanas S, Linderman RE, Chen FK, Carroll J. Assessing the use of incorrectly scaled optical coherence tomography angiography images in peer-reviewed studies: a systematic review. *JAMA Ophthalmol*. 2020; 138: 86–94. 31774456 10.1001/jamaophthalmol.2019.4821

